# Single Session and Short-Term Exercise for Mental Health Promotion in Tertiary Students: A Scoping Review

**DOI:** 10.1186/s40798-021-00358-y

**Published:** 2021-10-11

**Authors:** Michaela C. Pascoe, Alan P. Bailey, Melinda Craike, Tim Carter, Rhiannon K. Patten, Nigel K. Stepto, Alexandra G. Parker

**Affiliations:** 1 Institute for Health and Sport, Victoria University, Melbourne, VIC 3011 Australia; 2 Peter MacCallum Cancer Centre, 305 Grattan St, Melbourne, VIC 3000 Australia; 3 Orygen, The National Centre of Excellence in Youth Mental Health and Centre for Youth Mental Health, University of Melbourne, Melbourne, VIC 3010 Australia; 4 Mitchell Institute, Victoria University, Melbourne, VIC 3011 Australia; 5 Institute of Mental Health, School of Health Sciences, University of Nottingham, Triumph Road, Nottingham, UK; 6 Monash Centre for Health Research and Implementation, Monash University, Clayton, VIC 3800 Australia; 7 Australian Institute for Musculoskeletal Science, Victoria University, St Albans, VIC 3021 Australia; 8 Medicine-Western Health, Faculty of Medicine, Dentistry and Health Science, Melbourne University, Sunshine, VIC 3020 Australia

**Keywords:** Adolescent, Mental health, Exercise, Well-being, Physical activity

## Abstract

Exercise can improve mental health; however many tertiary students do not reach recommended levels of weekly engagement. Short-term exercise may be more achievable for tertiary students to engage in to promote mental health, particularly during times of high stress. The current scoping review aimed to provide an overview of controlled trials testing the effect of short-term (single bout and up to 3 weeks) exercise across mental health domains, both at rest and in response to an experimentally manipulated laboratory stress task, in tertiary students. The search was conducted using ‘Evidence Finder,’ a database of published and systematic reviews and controlled trials of interventions in the youth mental health field. A total of 14 trials meet inclusion criteria, six measured mental health symptoms in response to an experimentally manipulated laboratory stress task and the remaining eight measured mental health symptoms. We found that short-term exercise interventions appeared to reduce anxiety like symptoms and anxiety sensitivity and buffered against a drop in mood following an experimentally manipulated laboratory stress task. There was limited available evidence testing the impacts of exercise on depression like symptoms and other mental health mental health domains, suggesting further work is required. Universities should consider implementing methods to increase student knowledge about the relationship between physical exercise and mental health and student access to exercise facilities.

## Key Points

- Short-term exercise interventions reduce anxiety like symptoms and anxiety sensitivity in tertiary students.
- Short-term exercise interventions buffer against a drop in mood following an experimentally manipulated laboratory stress task in tertiary students.
- There was limited available evidence testing the impacts of exercise on depression like symptoms and other mental health symptoms in tertiary students.

## Introduction

Students in tertiary education settings face a wide range of ongoing normative stressors, which can be defined as normal day to day hassles such as ongoing academic demands. Accordingly, tertiary (defined here as post-secondary education) [1] students commonly experience ongoing stress relating to their education, such as pressure to achieve high marks [2]. Stress is a clear precipitant of a wide range of mental illnesses [3, 4] and the onset of mental illness is most likely to occur during adolescence and young adulthood [5]. Therefore, interventions that promote mental health as well as target the early phases or sub-threshold symptoms are urgently required [6].

Access to facilities and programs in tertiary settings that promote mental health has the potential to improve the mental health and functioning of young people and prevent the onset and the negative impacts of mental illness [7]. Exercise is defined as the planned, structured and repetitive undertaking of physical activity for the purposes of maintaining or improving health or skill-related components of physical fitness [8, 9]. Our previous work demonstrates that exercise can improve mental health in young people [10]; however many young people fail to reach the weekly recommended levels of exercise participation [11–13]. Disengagement from exercise, physical activity and sporting clubs occurs during adolescence [14, 15], with more than 80% of adolescents and 25% of adults worldwide being insufficiently active [16]. When individuals transition from secondary school to tertiary education, they can experience a further decline in exercise levels [17–19].

### Rationale

Short-term exercise is defined as any exercise intervention that is shorter than 3 weeks in duration [20, 21]. Short-term exercise may be an effective way to protect against mental health concerns in tertiary education settings, including during periods of high stress, such as exam periods [22]. Meta-analyses and systematic reviews of all populations and age groups show that short-term exercise, including a single bout of exercise, can reduce symptoms of anxiety [23] and increase feelings of energy [24], while in healthy adults single bouts of exercise increase performance on memory tasks [25]. The potential of short-term exercise as a mental health promotion strategy in tertiary students, specifically, remains unknown.

### Objectives

The review addressed the research question: What is known about the impact of short-term exercise for mental health promotion in tertiary students? ‘Mental health’ encompasses mental health symptoms as collected using quantitative outcome measures of mental ill-health symptom severity as well indicators of mental health, including well-being and quality-of-life [26]. We provide an overview of controlled trials that assess the effect of short-term exercise on mental health, in tertiary students.

The objectives were:

1. Examine the range/extent of outcomes from short-term exercise interventions on mental health in tertiary students;
2. Collate mental health data and provide an overview of the effect of short-term exercise on mental health in tertiary students.

## Methods

### Protocol/Registration

This review was reported and conducted in line with PRISMA-ScR guidelines for scoping reviews [27]. A review protocol was not registered or published.

### Eligibility Criteria

Study eligibility criteria were as follows: the sample comprised of tertiary students (mean age under 25.9 years); studies were published between 1980 and 2019 [28]; included an exercise intervention and a comparison condition; the exercise intervention/s lasted less than 3 weeks in duration [20, 21]; study designs were randomised controlled trials [RCTs] or non-randomized controlled trials and published in English and reported on at least one of the following mental health outcomes:

- Depression symptoms;
- Anxiety symptoms, or trait anxiety measures;
- Substance use or symptoms;
- Bipolar symptoms;
- Eating disorder symptoms;
- Trauma or stressor-related symptoms;
- General psychological distress;
- Wellbeing/functioning: Quality of life; Functioning (social, educational, vocational, employment).

Studies were excluded if: they were unpublished or comprised of a population diagnosed with a mental disorder or illness. This is because we have reviewed exercise and physical activity as a treatment for mental illness in our companion paper [29].

### Information Sources

Searches were conducted using ‘Evidence Finder’ (www.orygen.org.au). ‘Evidence Finder’ is a database of published systematic reviews and controlled trials of interventions delivered for youth mental health [28, 30]. The ‘Evidence Finder’ is a Federally funded, initiative designed to reduce duplication in method to enable more rapid translation of research evidence. It aims to facilitate evidence-based practice by providing a comprehensive summary of the extent and distribution of existing research. It achieves this through a rigorous process by which intervention trials and systematic reviews published in the area of youth mental health are systematically searched for and compiled into a central resource. It offers rapid access to the best available evidence and the vast coverage offered by the evidence mapping methodology provides an overall snapshot of where evidence exists and where it is lacking. Therefore, this easy access to the current body of evidence can inform and guide new research agendas towards addressing the gaps in our knowledge of intervention for mental health promotion among young people

In order to comprehensively and systematically assemble and appraise evidence for short-term physical activity across numerous mental-health outcomes, a mapping methodology was selected.

The database is populated annually using systematic searches of the following databases: Embase, PsycINFO, Cochrane Library and MEDLINE (see [28, 30] for detailed methodology). It includes prevention, treatment and relapse‐prevention studies. It also includes interventions delivered to young people (6–25 years), published between 1980 and 2019 and assessing the following mental health symptoms: bipolar, depression, anxiety, eating disorders, suicide-self harm, substance use and psychosis. It contains RCTs and quasi‐randomised studies as well as systematic reviews and/or meta‐analyses, published in English.

The following criteria were applied to the ‘Evidence Finder’ search engine: (1) ‘all’ mental health/substance use problems; (2) ‘all’ stages of illness; (3) ‘complementary and alternative interventions’ treatment/interventions, followed by ‘Physical activity/exercise’ (4) ‘all’ for Publication Date; (5) and ‘non’ for keywords. Advanced options were also selected to include randomized/ controlled clinical trials and systematic reviews.

### Search

A single author [MP] conducted a search of the literature using the ‘Evidence Finder’ in July 2018 (updated in May 2020). All studies that had been classified within ‘Evidence Finder’ as ‘Physical activity/Exercise’ and published between 1980 and 2019 were assessed. No other restrictions were applied to the ‘Evidence Finder’ search as the scoping review sought to synthesise a large number of outcomes across multiple mental health concerns. Moreover, the reference lists of identified literature reviews, systematic reviews and meta-analyses were searched for suitable primary research. In addition the studies identified using ‘Evidence Finder,’ the reference lists of reviews, systematic reviews, and meta-analyses retrieved from the ‘Evidence Finder’ were also checked to identify additional relevant studies.

### Selection of Sources of Evidence

Two authors independently undertook title and abstract screening (MP, AP). All full texts were reviewed independently by at least two authors (MP, AP, MC). There were no conflicts requiring resolution by a third author.

### Data Charting Process

Data charting [31] were undertaken by one author (MP) using the extraction forms shown in Tables 1 and 2. All extracted data were checked for accuracy by a second author (TC, AB). Data were obtained only and directly from the articles.

**Table 1:** Characteristics of included studies

Outcome assessed	Resting/induced	References	Country	Setting	Study design (groups)	Participants	Findings	Mechanism findings	PA/E groups	Non PA/E groups	Assessment timepoint	ITT	Resting/induced
Affect	Laboratory induced: image viewing	Bartholomew [32]	USA	University	RCT (2)	University students (*n* = 40) (*M* age = 23.4 yrs)	RET reduced negative imagery inducted decreases in positive affect at 45 m post exercise	None	RET (*n* = 17)	No intervention (*n* = 18)	Pre-post	No	Image viewing
Affect	Laboratory induced: memory task	Hopkins et al. [33]	USA	Community and University	RCT (4)	Individuals with a sedentary lifestyle (*n* = 75) (*M* age = 20.6 yrs)	No effect	4wks ET* + AET improved object recognition memory only in participants homozygous for the BDNF Val allele. Exercise-induced changes in cognition were not correlated with changes in mood/anxiety	4wks ET*: (Walking/jogging) (*n* = 14); 4wks ET* + AET (Walking/jogging) (*n* = 12); AET (Walking/jogging) (*n* = 15)	No intervention (*n* = 13)	Pre-post	NS	Memory task
Anger	Laboratory induced: image viewing	Bartholomew [32]	USA	University	RCT (2)	University students (*n* = 40) (*M* age = 23.4 yrs)	RET increased anger 5 m post exercise	None	RET (*n* = 17)	No intervention (*n* = 18)	Pre-post	No	Image viewing
Anxiety	Laboratory induced: image viewing	Bartholomew [32]	USA	University	RCT (2)	University students (*n* = 40) (*M* age = 23.4 yrs)	RET increased anxiety 5 m and decreased anxiety at 30 and 45 m post exercise. RET decreased negative imagery induced anxiety at 15, 30 and 45 m post exercise	None	RET (*n* = 17)	No intervention (*n* = 18)	Pre-post	No	Image viewing
Anxiety	Laboratory induced: memory task	Hopkins et al. [33]	USA	Community and University	RCT (4)	Individuals with a sedentary lifestyle (*n* = 75) (*M* age = 20.6 yrs)	No effect	4wks ET* + AET improved object recognition memory only in participants homozygous for the BDNF Val allele. Exercise-induced changes in cognition were not correlated with changes in mood/anxiety	4wks ET*: (Walking/jogging) (*n* = 14); 4wks ET* + AET (Walking/jogging) (*n* = 12); AET (Walking/jogging) (*n* = 15)	No intervention (*n* = 13)	Pre-post	NS	Memory task
Anxiety	Laboratory induced: speaking challenge	Julian et al. [34]	USA	University	RCT (4)	Individuals with elevated self-reported anxiety like symptoms (*n* = 112) (*M* age = 19.9 yrs)	No effect of exercise or attention training on anxiety reactivity	No effect of exercise or attention training on attention bias	Walking/jogging w. attention modification program (*n* = 28); Walking/jogging w/o attention modification program (*n* = 28)	Rest with Attention Modification Program (*n* = 28); Rest without Attention Modification Program (*n* = 28)	Pre-post, FU (4mths)	NS	Speaking challenge
Anxiety	Resting	Mothes et al. [35]	Germany	Community and University	RCT (4)	Inactive individuals (*n* = 76) (*M* age = 21.89 yrs)	AET (all groups combined) decreased state anxiety (PP). No difference between groups	State-anxiety reduced more during AE in participants with greater habitual exercise expectations	AET + enhanced expectation (*n* = 18) AET + expectation (*n* = 20) AET (*n* = 19) AET + no-effect expectation (*n* = 19) (all cycling ergometer)	None	Pre-post	NS	Resting
Anxiety	Resting	Smits et al. [36]	USA	Community and University	RCT (3)	University students scoring ≥ 25 on the ASI (*n* = 60) (*M* age = 20.7 yrs)	AET and AET + cognitive restructuring reduced anxiety (BG)	Changes in anxiety sensitivity mediated the beneficial effects of exercise on anxious and depressed mood	AET (*n* = 19); AET + cognitive restructuring (*n* = 21)	WL (*n* = 20)	Pre-post	Yes	Resting
Anxiety	Resting	Lindheimer et al. [37]	USA	University	RCT (4)	University students with a raw score of 40 or higher on form Y-2 of the State Trait Anxiety Inventory (STAI) (*n* = 60) (*M* age = 20.3 yrs)	No effect	Cycling did not affect energy and working memory after a single session	AET-cycling + info (*n* = 15); AET-cycling (*n* = 15)	Cycling placebo + info (*n* = 15); Cycling placebo (*n* = 15)	Pre-post	NS	Resting
Anxiety	Resting	Broman-Fulks et al. [38]	USA	University	RCT (2)	University students scoring ≥ 25 on the ASI (*n* = 54) (*M* age = 21.2 yrs)	AET-high reduced state anxiety (BG)	None	AET-high (*n* = 29); AET-low (*n* = 25)	None	Pre-post, FU (1wk)	NS	Resting
Anxiety	Resting	Focht et al. [39]	USA	University	RCT (3)	Female university students (*n* = 54) (*M* age = 21.2 yrs)	RET-mod (circuit) reduced state anxiety (BG)	RET-high (multiple set) increased body awareness and SBP	RET-mod (circuit) (*n* = ns); RET-high (multiple set) (*n* = ns)	Video watching (*n* = ns)	Pre-post	NS	Resting
Anxiety sensitivity	Resting	Mason and Asmundson [40]	USA	Community and University	RCT (3)	Inactive individuals (*n* = 63) (*M* age = 24.63 yrs)	Both AET sprint-interval and AET continuous reduced anxiety sensitivity compared to waitlist at post-test (BG). No effect at FU	None	AET sprint-interval (*n* = 16) AET continuous (*n* = 20)	WL (*n* = 20)	Pre-post (FU 3 and 7 days)	NS	Resting
Anxiety sensitivity	Resting	Medina et al. [41]/Smits et al. [42]	USA	Community and University	RCT (3)	University students scoring ≥ 25 on the ASI (*n* = 60) (*M* age = 20.7 yrs)	AET decreased anxiety sensitivity (BG); AET and AET + cognitive restructuring (combined) reduced anxiety sensitivity (BG)	Changes in anxiety sensitivity mediated effects of exercise on anxious and depressed mood. Males showed reductions in anxiety sensitivity sooner than females	AET (*n* = 19); AET + cognitive restructuring (*n* = 21)	WL (*n* = 20)	Pre-post	Yes	Resting
Anxiety sensitivity	Resting	Broman-Fulks et al. [38]	USA	University	RCT (2)	University students scoring ≥ 25 on the ASI (*n* = 54) (*M* age = 21.2 yrs)	AET-high and AET-low reduced anxiety sensitivity (PP). AET-high caused more rapid reductions in anxiety sensitivity (BG)	None	AET-high (*n* = 29); AET-low (*n* = 25)	None	Pre-post, FU (1wk)	NS	Resting
Anxiety sensitivity	Resting	Broman-Fulks and Storey [43]	USA	University	RCT (2)	University students scoring > 26 on the ASI-R (*n* = 35) (*M* age = 18.9 yrs)	AE reduced anxiety sensitivity (BG)	None	AE (*n* = 19)	No intervention (*n* = 16)	Pre-post, FU (1wk)	No	Resting
Depression	Laboratory induced: memory task	Hopkins et al. [33]	USA	Community and University	RCT (4)	Individuals with a sedentary lifestyle (*n* = 75) (*M* age = 20.6 yrs)	No effect	4wks ET* + AET improved object recognition memory only in participants homozygous for the BDNF Val allele. Exercise-induced changes in cognition were not correlated with changes in mood/anxiety	4wks ET*: (Walking/jogging) (*n* = 14); 4wks ET* + AET (Walking/jogging) (*n* = 12); AET (Walking/jogging) (*n* = 15)	No intervention (*n* = 13)	Pre-post	NS	Memory task
Depression/anxiety	Resting	Mothes et al. [35]	Germany	Community and University	RCT (4)	Inactive individuals (*n* = 76) (*M* age = 21.89 yrs)	AET (all groups combined) decreased depression/anxiety (PP). No difference BG	Habitual exercise expectations were not correlated with changes in anxiety/depression during exercise	AET + enhanced expectation (*n* = 18) AET + expectation (*n* = 20) AET (*n* = 19) AET + no-effect expectation (*n* = 19) (all cycling ergometer)	None	Pre-post	NS	Resting
Depression	Resting	Smits et al. [36]	USA	Community and University	RCT (3)	University students scoring ≥ 25 on the ASI (*n* = 60) (*M* age = 20.7 yrs)	AET and AET + cognitive restructuring reduced depression (BG)	Changes in anxiety sensitivity mediated the beneficial effects of exercise on anxious and depressed mood	AET (*n* = 19); AET + cognitive restructuring (*n* = 21)	WL (*n* = 20)	Pre-post	Yes	Resting
Distress tolerance	Resting	Mason and Asmundson [40]	USA	Community and University	RCT (3)	Inactive individuals (*n* = 63) (*M* age = 24.63 yrs)	No effect	None	AET sprint-interval (*n* = 16) AET continuous (*n* = 20)	WL (*n* = 20)	Pre-post (FU 3 and 7 days)	NS	Resting
Eating disorder symptoms	Resting	Fallon and Hausenblas [44]	USA	University	RCT (2)	Female university students with high drive for thinness (*n* = 63) (*M* age = 19.8 yrs)	No effect	None	AET (*n* = 32)	Rest (*n* = 31)	Pre-post	NS	Resting
Mood states	Resting	Lindheimer et al. [37]	USA	University	RCT (4)	University students with a raw score of 40 or higher on form Y-2 of the State Trait Anxiety Inventory (STAI) (*n* = 60) (*M* age = 20.3 yrs)	No effect	Cycling did not affect energy and working memory after a single session	AET-cycling + info (*n* = 15); AET-cycling (*n* = 15)	Cycling placebo + info (*n* = 15); Cycling placebo (*n* = 15)	Pre-post	NS	Resting
Mood states	Resting	Fallon and Hausenblas [44]	USA	University	RCT (2)	Female university students with high drive for thinness (*n* = 63) (*M* age = 19.8 yrs)	No effect	None	AET (*n* = 32)	Rest (*n* = 31)	Pre-post	NS	Resting
Mood states	Laboratory induced: cognitive task	Roth [45]	USA	University	RCT (2)	Active and inactive university students (*n* = 80) (*M* age = 20.8 yrs)	AET improved mood states (tension-anxiety; confusion-bewilderment)	Acute reductions in anxiety following single bouts of exercise occurred in the absence of changes in cardiovascular reactivity	AET (*n* = 40)	WL (*n* = 40)	Pre-post	NS	Cognitive task
Mood states	Laboratory induced: cognitive task and resting	Roth et al. [46]	USA	University	RCT (2)	Female university students (*n* = 57) (*M* age = 20.5 yrs)	AET improved mood states (tension/anxiety; vigour/activity) in acute and basal conditions	AET had vasodilative effects	Exercise/stress task (*n* = 15); exercise/no stress task (*n* = 14)	Rest/stress task (*n* = 14); rest/no stress task (*n* = 14)	Pre-post	NS	Cognitive task and resting
Stress	Laboratory induced: memory task	Hopkins et al. [33]	USA	Community and University	RCT (4)	Individuals with a sedentary lifestyle (*n* = 75) (*M* age = 20.6 yrs)	4wks ET* + AET decreased stress. AET increased stress	4wks ET* + AET improved object recognition memory only in participants homozygous for the BDNF Val allele. Exercise-induced changes in cognition were not correlated with changes in mood/anxiety	4wks ET*: (Walking/jogging) (*n* = 14); 4wks ET* + AET (Walking/jogging) (*n* = 12); AET (Walking/jogging) (*n* = 15)	No intervention (*n* = 13)	Pre-post	NS	Memory task

*AET* aerobic exercise training, *AET-high* aerobic exercise training low intensity, *AET-low* aerobic exercise training low intensity, *ASI* Anxiety Sensitivity Index, *ASI-R* Anxiety Sensitivity Index-Revised, *BDNF* brain-derived neurotrophic factor, *BG* between group difference, *FU* follow-up, *ITT* intention to treat analysis, *LIET* low intensity exercise training, 4wks ET* 4 weeks of walking or jogging on a treadmill—impact of this arm of the study not reported in text as does not meet inclusion criteria that intervention should be less than 3 weeks in duration, *mths* months, *M* mean, *m* minutes, *n* sample size, *PP* pre-post intervention, *RCT* randomised controlled trial, *RET* resistance exercise training, *RET-mod* resistance exercise training moderate intensity, *RET-high* resistance exercise training high intensity, *SBP* systolic blood pressure, *wk* week, *WL* waitlist, *yrs* years

**Table 2:** Characteristics of the interventions

Study	PA/exercise interventions	Exercise intensity/type	Non PA/exercise interventions	Personnel delivering treatment	Individual/group	Duration frequency	Stress condition
Bartholomew [32]	RE: "participants performed sets of 4 reps for each of 6 exercises. They were asked to rate the intensity of each set using Borg's 6–19 RPE scale. The level of resistance for each set was manipulated until an RPE equal to 15 (hard) was reported. The level of resistance was noted and used as the intensity for the experimental resistance exercise sessions."	RE: VIGOROUS, RESISTANCE	Placebo: "Placebo activity participants were shown copies of college yearbook photographs and were asked to provide a detailed personality profile for as many individuals as they could within 20 min"	Researcher	Individual	20 m single session	Laboratory induced
Broman-Fulks et al. [38]	AE-high: (walking/jogging) HIET: "2 min of stretching exercises and a 2 min treadmill warm-up then briskly walk or jog on a treadmill at a speed that produced exercise HR between 60–90% of the individual’s age adjusted predicted MHR. Treadmill speed was adjusted as necessary" AE-low: (walking/jogging) LIET: "The comparison group completed a similar protocol except treadmill speed was maintained at 1-mile per hour so participant heart rate never reached 60% of MHR"	AE-high: VIGOROUS (Aerobic); AE-low: LIGHT to MODERATE	NA	Researcher	Individual	20 m 2-4xwk/2wks	Resting
Broman-Fulks and Storey [43]	AE: (walking/jogging) "Exercise participants were asked to briskly walk or jog on a treadmill at a speed that maintained their HR between 60–90% of their predicted MHR for the full 20-min session. Treadmill speeds were adjusted as necessary. Following each exercise session, participants completed a 5-min cool down"	AE: MODERATE to VIGOROUS, AEROBIC	"Individuals assigned to the no-exercise control condition reported to the lab 6 times over 2 weeks (no fewer than 2 and no more than 4 times per week) just to complete the ASI-R."	A researcher	Not stated	20 m 2-4xwk/2wks	Resting
Fallon and Hausenblas [44]	AE: "Following the 5 min warm-up period, participants self-selected a speed and incline on the treadmill that would create a ‘‘moderate intensity’’ exercise bout. After 20 min of moderate intensity exercise, the participants were asked to slow to a mild walking pace for a 5 min cool down"	AE: MODERATE to VIGOROUS, AEROBIC	Rest: "The participants were allowed to read or sit quietly for 30 min. Study materials were checked to confirm that they did not discuss diet, exercise, or pictorially depict an ideal body type"	Not stated	Individual	30 m single session	Resting
Focht et al. [39]	RE-mod: "(weight training) circuit: a single set of 10 to 20 reps at 50% of 1 RM for each exercise while utilizing 30 to 45 s recovery interval between each set and exercise" RE-high: "(weight training) multiple set: 3 sets of 6 to 10 reps at 75% of 1 RM for each exercise while utilizing a 1 to 2 min recovery interval between each set and exercise"	RE-mod: MODERATE, RESISTANCE RE-high: VIGOROUS, RESISTANCE	Video: "watched a videotape of resistance exercise techniques that was of comparable length to the resistance exercise sessions"	Not stated	Not stated	30 m single session	Resting
Hopkins et al. [33]	AE (4x/wk-): Walk or jog continuously on a treadmill for a minimum of 30 min, at a minimum speed of 3.5 mph (equivalent to a brisk walk) four times per week (no acute session on final day) AE (4x/wk+): Walk or jog continuously on a treadmill for a minimum of 30 min, at a minimum speed of 3.5 mph four times per week, plus a single acute session on the final day of the study Acute session: A single session on the final day of the study (walk or jog continuously on a treadmill for a minimum of 30 min, at a minimum speed of 3.5 mph)	AE: CBD [likely MODERATE to VIGOROUS], AEROBIC Acute: CBD [likely MODERATE to VIGOROUS], AEROBIC	No intervention	Not stated	Not stated	AE: 30 m 4xwk/4wks; Acute session: 30 m single session	Laboratory induced
Julian et al. [34]	AE: (walking/jogging) "Supervised, moderate-intensity (65–70% MHR) treadmill exercise. Exercise began with a 5 min warm-up at progressively increasing speed. Participants then trained at the target heart rate for 20 min, after which they completed a 5 min cool-down, during which the speed was gradually reduced, and participants then stretched"	AE: MODERATE, AEROBIC	Rest: "Participants assigned to the rest condition rested for 30 min"	Not stated (states were supervised)	Not stated	30 m single session	Laboratory induced
Lindheimer et al. [37]	Cycling: participants in the active condition pedalled at an intensity of 35% VO2 reserve for a duration of 25 min and cadence of 55 revolutions per minute on a semi-recumbent cycle ergometer (RT-300-SL; Restorative Therapies Inc)	Light, aerobic	Participants in the passive cycling condition had their legs moved at a constant speed (55 revolutions per minute) via a motorized pedalling system for 30 min to control for the confounding effects of context and leg movement experienced during active cycling	Not stated	Not stated	30 m single session	Resting
Mason and Asmundson [40]	AE-sprint interval: a 2-min warm-up, consisting of low intensity cycling (i.e., generating less than 50 W), followed by three 20-s cycle sprints against an applied resistance at an intensity at or above 18 RPE and 85% of their age adjusted HR max, and separated by an active recovery consisting of approximately 2 min of low intensity cycling. The SIT session was followed by a 3-min cool-down, consisting of low intensity cycling AE-continuous: participants underwent a 2-min warm up consisting of low intensity cycling (i.e., generating less than 50 W) followed by 45 min of MICT on a stationary spin cycle, then a 3-min cool down. Participants maintained a heart rate of 70% of their estimated age adjusted HR max and an RPE between 13 and 15 throughout the 45 min of training	AE-sprint interval: VIGOROUS, AEROBIC AE-continuous: MODERATE to VIGOROUS, AEROBIC	No intervention	Certified personal trainer	Not stated	AE-sprint interval: 10 m single session AE-continuous: 50 m single session	Resting
Medina et al. [41]/Smits et al. [42]	AE: "Moderate intensity aerobic exercise sessions at the laboratory in a room furnished with treadmills and appropriate safety equipment. Exercise intensity was pre-set at the high-moderate level (70% of HR max). The experimenter adjusted the speed and incline of the treadmill to target specific levels of effort as assessed by HR"	AE: MODERATE to VIGOROUS, AEROBIC	WL	Certified study personnel	Individual	20 m 3xwk/2wks	Resting
Mothes et al. [35]	AE: 2 min warm up and 30 min of cycling on an ergometer at approximately 75 RPM at 40% max power output AE+ expectation: same as AE plus participants viewed a multimedia presentation that aimed to induce positive outcome expectations regarding the subsequent exercise (‘‘According to recent research this exercise is perfectly suitable for improving your immediate well-being’’) AE+ enhanced expectations: same as AE plus viewing the multimedia presentation that aimed to induce positive outcome expectations regarding the subsequent exercise and aiming to induce an even stronger expectation by additionally focusing on the compression shirt lent to all participants to wear during exercise (‘‘Wearing this shirt will enhance physical capacity and increase exercise benefits’’) AE no-effect expectation: same as AE plus viewing multi-media presentation aimed to induce a more neutral outcome expectation (‘‘According to recent research this exercise is not suitable for improving your immediate well-being, since it is too short and too weak’’)	AE: MODERATE, AEROBIC	NA	Researchers	Not stated	30 m single session	Resting
Roth [45]	AE: (cycling) The exercise condition involved supervised exercise on a Bodyguard 990 bicycle ergometer. The subject pedalled in time with a metronome to achieve a pedalling rate of 50 revolutions per minute. The initial workload was set at 600 kilopond meters per minute (kpm/min) for males and 300 kpm/min for females. For the remainder of the 20-min period. the workload was decreased, if necessary, to maintain a steady exercise heart rate between 115 and 135 bpm, verified by heart rate measurements obtained every 3 min	AE: MODERATE, AEROBIC	No intervention	Research personnel	Not stated	20 m single session	Laboratory induced
Roth et al. [46]	AE: (cycling) Subjects mounted a Bodyguard 990 bicycle ergometer and pedalled in time with a metronome to achieve a pedalling rate of 50 revolutions per minute. 1 kilopond of resistance was applied, resulting in a workload of 300 kilopond meters per min (50 W)	AE: CBD [reported as LIGHT to MODERATE], AEROBIC	Rest: subjects assigned to the no-exercise condition were instructed to simply remain seated for several minutes until given further instructions	Not stated	Individual	10 m single session	Laboratory induced
Smits [36]	AE: Participants in the exercise condition completed exercise at 70% of HR max on a treadmill. The training program consisted of a 3 min warm-up at a progressively increasing speed until 70% of HR max was reached. Participants then trained at that target heart rate for 20 min	AE: MODERATE to VIGOROUS, AEROBIC	Rest: participants in the quiet rest condition sat quietly for 23 min	Not stated	Not stated	20 m single session	Laboratory induced

*1RM* 1 repetition maximum, *AE* aerobic exercise, *ASI-R* Anxiety Sensitivity Index-Revised, *bpm* beats per minute, *CBD* cannot be determined, *E* exercise, *High* high intensity, *HIET* high intensity exercise training, *HR* heart rate, *LIET* low intensity exercise training, *Low* low intensity, *M/Mins* minutes, *MOD* moderate, *MHR* maximum heart rate, *mph* miles per hour, *NA* not applicable, *NS* not stated, *PA* physical activity, *PE* physical education, *RE* resistance exercise, *Reps* repetitions, *RPE* rating of perceived exertion, *TAU* treatment as usual, *VIG* vigorous, *VO2* oxygen consumption, *Wk* week, *WL* wait list

Two assessors (NS, RP) reviewed studies for subjective (perceived exertion ratings) and objective measures of exercise intensity (heart rates [HR], %maximal HR, %HR reserve, %1-repetition maximum, percent of maximal-oxygen-uptake [%VO_2max_]). Using these, the interventions were classified by intensity: (1) light-intensity, (2) light-to-moderate-intensity, (3) moderate-intensity, (4) moderate-to-vigorous-intensity, or (5) vigorous-intensity for resistance exercise [47] and aerobic exercise [21]. If possible, we attempted to estimate intensity based on the compendium of exercise energy expenditure, where the interventions were insufficiently described [48]. We did not contact authors for further information when insufficient information was provided in the published articles.

### Data Items

The following data were extracted: study design type, setting of study, country of study origin, sample size, participants and mean age, tools employed to measure mental health domain, the mental health domain assessed, assessment time points, overall findings, and if intention to treat analysis was used (Table 1). We also extracted characteristics of the exercise intervention and control intervention, including the delivery format, duration and frequency of the intervention and what personnel delivered the intervention (Table 2).

### Critical Appraisal of Individual Sources of Evidence

As scoping reviews are generally conducted to provide an overview of the existing evidence regardless of methodological quality or risk of bias, the included sources of evidence are typically not critically appraised, as per PRISMA-ScR guidelines [27], and we, did not conduct a risk of bias assessment.

### Synthesis of Results

We used the mental health domain as the common analytical framework in our ‘descriptive-analytical’ approach [31]. The combination of exercise intensity (light, moderate or vigorous) and duration (minutes/week) determines the session and intervention dose of exercise interventions. We focused on exercise intensity rather than dose, due to heterogeneity of the duration of interventions and given that exercise intensity is strongly linked to affective responses and sustainability [49, 50].

## Results

### Selection of Sources of Evidence

**Fig. 1:**
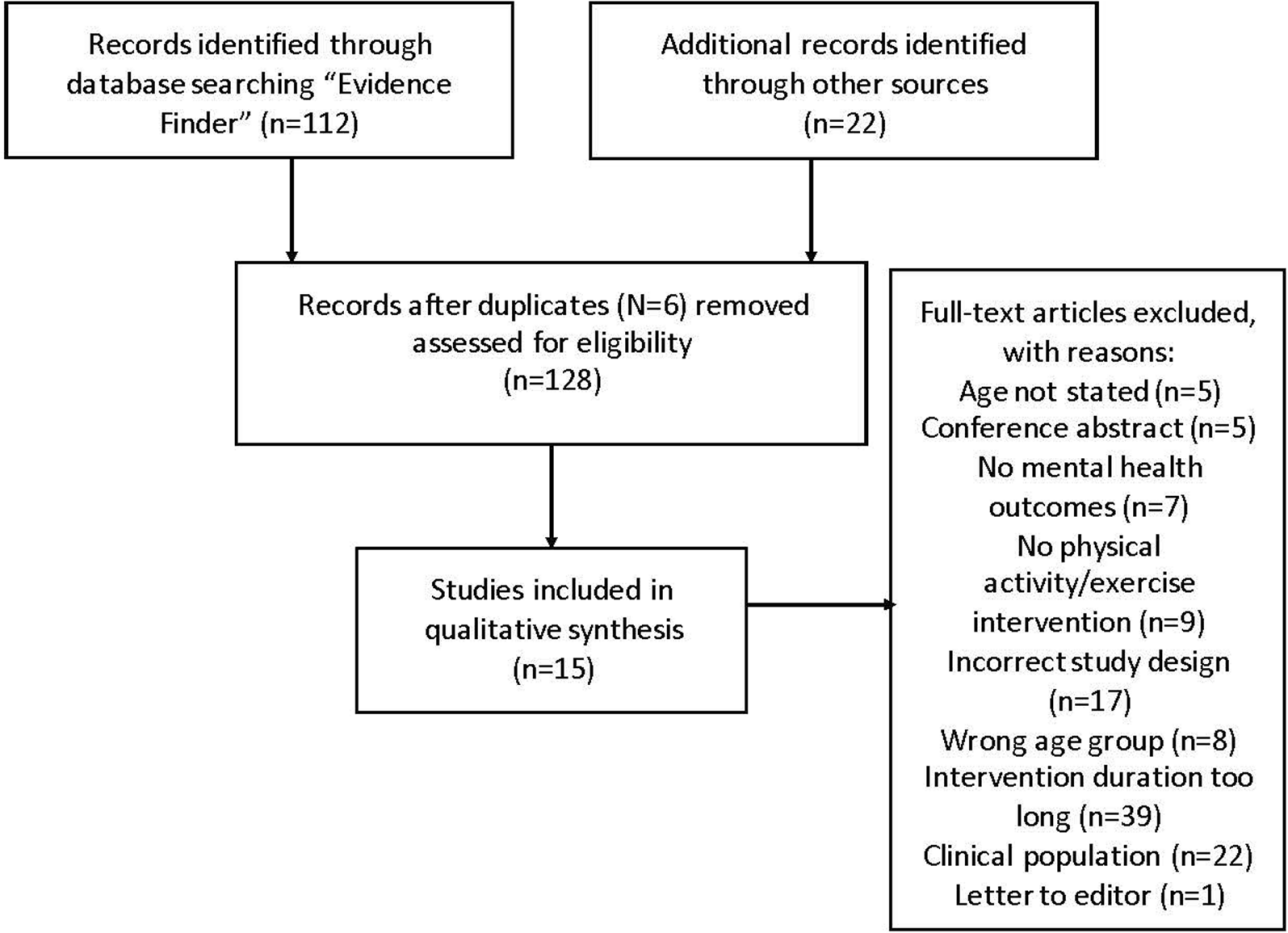
PRISMA flow diagram showing reasons for study exclusions

A total of 112 records were returned using the ‘Evidence Finder.’ Additional articles (*n* = 22) were searched for and identified by going through the retrieved searching reviews, systematic reviews, and meta-analyses identified through ‘Evidence Finder’ (Fig. 1). In total, 15 studies were included; however two of these studies [41, 42] report different outcomes from the same trial and therefore were combined in this review, resulting in inclusion of 14 studies.

### Characteristics of Sources of Evidence

Six of these studies measured mental health symptoms in response to an experimentally manipulated laboratory stress task [32–34, 42, 45, 46], and the remaining eight measured mental health [35, 37–44]. Ten studies delivered a single bout of exercise [32, 34–37, 39, 40, 44–46] and four delivered short-term (under 3 weeks) exercise sessions or programs [33, 38, 41–43]. All studies were RCTs. Seven of the 12 studies included as an eligibility criteria young people with elevated symptomology at baseline; two with elevated anxiety like symptoms [34, 37], four with high anxiety sensitivity or proneness [36, 38, 41–43], and one with elevated eating disorder symptoms [44]. In the remaining seven studies [32, 33, 35, 39, 40, 45, 46] tertiary students were not required to have elevated mental health symptoms for study inclusion.

### Results of Sources of Evidence

Extracted data are shown in Tables 1 and 2. As shown in Table 2, five interventions were delivered individually, and the remaining seven did not state the method of delivery.

### Types of Interventions

**Fig. 2:**
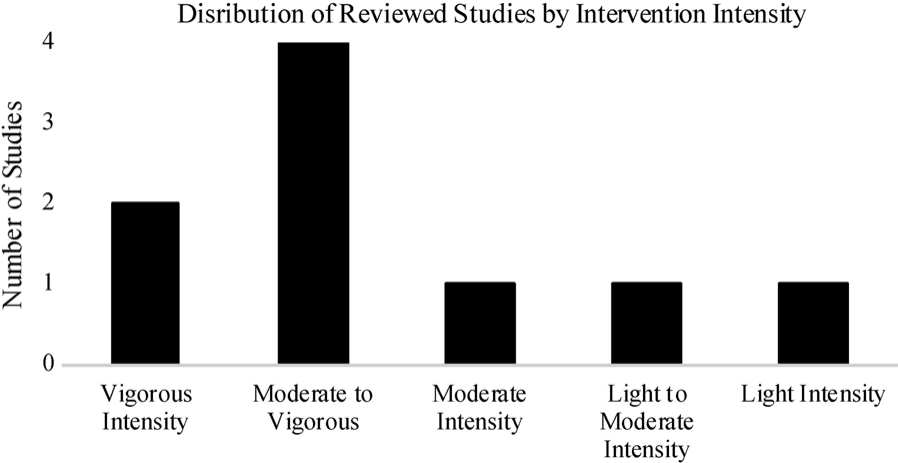
Distribution of interventions studied by intensity

Figure 2 shows that moderate to vigorous intensity exercise interventions were the most frequently studied, and were most commonly running/jogging on a treadmill. Vigorous intensity interventions were also commonly studied, and included resistance exercise and jogging/sprinting. Moderate intensity exercises were also examined, and included cycling, walking/jogging and resistance exercises.

### Synthesis of Results

#### Anxiety Like Symptoms

Five studies assessed the impact of short-term exercise on anxiety like symptoms [35–39], and 80% (4/5) indicated that exercise reduced anxiety like symptoms [35, 36, 38, 39]; however, one of these studies did not include a non-exercise-based control group [35]. In another of these studies, a 2-week-long vigorous intensity aerobic intervention reduced state anxiety compared to a light-to-moderate intensity intervention, in tertiary students with high proneness to anxiety [38]. In another study involving tertiary students with high proneness to anxiety, a single bout of moderate-to-vigorous intensity aerobic exercise decreased anxiety like symptoms, as compared to no intervention [36]. In the third study, a single bout of moderate-intensity resistance-based exercise reduced state anxiety, compared to video watching, in healthy female tertiary students [39]. The single study that found no influence of a light intensity aerobic intervention on anxiety like symptoms, compared exercise to involuntary, passive cycling movement, during which the participants' legs were moved using a passive motorized cycle [37].

#### Anxiety Proneness or Sensitivity

Three studies assessed anxiety proneness or sensitivity, defined as the fear of the sensations that accompany anxiety, such as elevated heart rate, and 100% (3/3) found that short-term exercise decreased anxiety sensitivity [38, 42, 43]. Two studies assessed the impact of moderate-to-vigorous intensity aerobic interventions, one reported that a 2-week-long intervention reduced anxiety sensitivity in tertiary students with high anxiety sensitivity scores, compared to no-intervention [43] and the other reported that a single bout of moderate-to-vigorous-intensity aerobic exercise reduced anxiety sensitivity, compared to a wait-list (WL) control [41, 42]. In one of these studies, changes in depressed and anxious mood were mediated by the effects of the single bout of exercise on anxiety sensitivity [42]. The third study reported that both a 2-week-long vigorous aerobic intervention and a 2-week-long light-to-moderate intensity intervention reduced anxiety sensitivity from pre-to-post intervention, but that the vigorous-intensity intervention resulted in a more rapid reduction in anxiety sensitivity in tertiary students who had high anxiety sensitivity. This study compared two exercise interventions however so it lacked a non-exercise control group [38].

#### Mood States

Two studies assessed mood states and neither found that exercise influenced mood states [37, 44]. One study found no effect of a single bout of moderate-to-vigorous-intensity aerobic exercise on mood states, compared to a rest condition, in female tertiary students with a high drive for thinness [44]. The remaining study similarly found no effect of a stand-alone session of light intensity aerobic exercise, compared to involuntary, passive cycling movement, during which the participants' legs were passively moved, using motorized cycle, among tertiary students with high anxiety like symptoms [37].

#### Depression Like Symptoms

Two studies assessed depression like symptoms [35, 42]*.* One study reported that a single bout of moderate-to-vigorous intensity aerobic exercise decreased depression like symptoms in tertiary students with elevated depression, anxiety and stress symptoms at baseline, as compared to a wait list control group [42]*.* The second study found that all four exercise interventions assessed depression like symptoms from pre- to post-intervention; however this study did not include a non-exercise-based control group [35].

#### Outcomes Assessed by a Single Study

Another study found that a single session of moderate-to-vigorous intensity aerobic exercise did not influence eating disorder symptoms in female tertiary students with a high drive for thinness, compared to rest [44]. A single bout of vigorous intensity resistance exercise increased anger at 5 min following exercise, compared to no intervention, in health tertiary students [32].

#### Mental Health Symptoms in Response to an Experimentally Manipulated Laboratory Stressful Task

##### Anxiety Like Symptoms Following an Experimentally Manipulated Laboratory Stressful Task

Three studies tested the impact of short-term exercise on anxiety like symptoms following a stressful task [32–34] and 33% (1/3) indicated that exercise reduced anxiety like symptoms. In one of these studies, a single session of vigorous-intensity resistance-based exercise increased self-reported anxiety like symptoms 5 min following exercise, but reduced anxiety like symptoms at 30 and 45 min following exercise, compared to a no-intervention control group, in healthy students [32]. There was no effect of a single aerobic exercise session on anxiety like symptoms following a memory task, compared to no intervention, in students with a sedentary lifestyle [33]. There was similarly no effect of a single bout of moderate-vigorous intensity aerobic exercise on anxiety like symptoms, following a speaking challenge, compared to rest, in individuals with elevated self-reported anxiety like symptoms [34].

##### Mood States Following an Experimentally Manipulated Stressful Task

Two studies assessed mood states in response to a laboratory stress task in the form of a test of mental ability (the digits backward test), and both found a single session of exercise prior to completing the stressful task improved mood states, in active and inactive tertiary students [45, 46]. A single session of moderate-intensity aerobic exercise improved mood states, as compared to wait list group, in the absence of any observed changes in cardiovascular reactivity [45], as did a single session of light-moderate aerobic exercise, compared to rest, in healthy female tertiary students [46].

#### Affect Following an Experimentally Manipulated Stressful Task

Two studies assessed affect following either recall and description of extremely upsetting experiences [32], or a recognition memory task [33]. One of these studies found that a single session of vigorous intensity resistance-based exercise following viewing of negative imagery, blunted decreases in positive affect, compared to no intervention, in healthy university students [32]. In the second study, a single aerobic exercise session had no effect on affect following a memory task, compared to no intervention, in tertiary students with a sedentary lifestyle [33].

#### Stress Following an Experimentally Manipulated Stressful Task

A single aerobic exercise session did not reduce stress or depression like symptoms, in response to a recognition memory task, in individuals with a sedentary lifestyle and compared to no intervention, in tertiary students with a sedentary lifestyle [33].

## Discussion

The finding of the current scoping review indicates that short-term exercise may be beneficial for mental health promotion among tertiary students. Therobicis is an important finding as short-term exercise may buffer against the detrimental effects of stress during periods of high stress in tertiary students who do not engage in regular exercise. Importantly, a previous meta-analysis demonstrated that lifestyle interventions targeting physical activity in tertiary settings significantly increased the amount of moderate physical activity undertaken by tertiary students, indicating that tertiary institutions are suitable settings for implementing such interventions to promote physical activity and exercise [51]. Short-term exercise may be more achievable for tertiary students to engage in, compared to achieving an ongoing exercise regime, to promote mental health, particularly during times of high stress.

### Summary of Evidence

#### Mental Health

The available evidence indicates that short-term exercise results in improvements in anxiety like symptoms [36, 38, 39] and anxiety sensitivity or proneness [38, 42, 43], but not mood states [37, 44]. This is consistent with our previous scoping reviews examining the effects of longer term (longer than 3 weeks) exercise and physical activity interventions on mental health [10]. In our earlier scoping review, we found that moderate-to-vigorous-intensity and light-intensity exercise and physical activity reduce anxiety like symptoms and improve mood states, in healthy young people and young people with sub-threshold mental health symptoms [10]. In individuals with a mental illness, we previously found that light-to-moderate intensity exercise reduces anxiety, and moderate-to-vigorous intensity exercise can improve mood states [29], which may be an important consideration in that immediate changes in mood states may increase motivation or adherence to continued engagement in exercise [52, 53]. In particular, the current findings are consistent with previous work showing that vigorous-intensity exercise, but not light-intensity exercise, can reduce remission rates and irritability and improve mood states, in women with generalised anxiety disorder, compared to a waitlist control group [54], but does not reduce depression, anxiety like symptoms or worry [55]. It is still unclear exactly why exercise may be beneficial for anxiety like symptoms in particular and this is a worthwhile area of future inquiry. There is limited evidence regarding the impact of short-term exercise on other mental health concerns, such as eating disorder symptoms and stress, indicating that these are areas that require further investigation.

#### Exercise and Mood States Following an Experimentally Manipulated Stressful Task

The available evidence indicates that a single bout of exercise can improve mood states in response to an experimentally manipulated laboratory stress task [45, 46], indicating that exercise may be beneficial for mood when students are coping with stressful situations such as exam pressures.

### Intervention Characteristics

There were too few studies identified in the current scoping review to determine if outcomes vary depending on intervention intensity or approach. A previous systematic review of interventions promoting physical activity among university students, however, concluded that interventions would do well to address a number of behavioural determinants and argued that personalised approaches should be considered [56]. These authors highlight that many exercise interventions delivered in tertiary settings are not individualised and do not assess the unique needs of participants such as motives to engage in exercise interventions, skills required and self-regulatory techniques [56]. It is unknown if such considerations are relevant to achieve adherence to and engagement with short-term exercise interventions, and this could be an area of future investigation.

## Strengths and Limitations

This scoping review has a number of strengths. It is the first to examine the effects of short-term exercise on mental health concerns and in response to an experimentally manipulated laboratory stress test, in young people in tertiary education settings. It was conducted in concordance with and reported according to PRISMA guidance. The review also has some limitations. Six studies compared exercise to a wait-list group or to an additional exercise group, rather than to a non-exercise-based comparison group [33, 35, 38, 40–42, 45]. Therefore, while these studies can contribute to the literature in terms of demonstrating if short term exercise can result in pre-post changes in mental health measures, and if different exercise types and intensities are associated with benefits, without a non-exercise-based control group, it is unknown if the observed effects result from the exercise intervention, or from non-specific factors other than the intervention, such as time/attention effects [57]. In the current study, the exercise interventions are described only in terms of intensity; however there may be other potentially important aspects other than intervention intensity that influence mental health symptoms, such as exercise duration, adherence and the specific method of exercise employed, which we have not considered. As scoping reviews are generally conducted to provide an overview of the existing evidence regardless of methodological quality or risk of bias, the included sources of evidence are typically not critically appraised, as per PRISMA-ScR guidelines; however without conducting a risk of bias assessment [58], the quality of the included studies is unknown.

## Future Directions

Some universities are embedding information of the relationship between mental health and physical activity into student mental health strategies, such as Victoria University, Melbourne which states that it aims to, ‘raise awareness amongst all staff and students of the enablers of good mental health and ensure easy access to further support and guidance on these enablers. Enablers can include sleep, diet/alcohol, accommodation, finance, sport, physical activity and study skills [59].’ Therefore, tertiary education settings might consider implementing methods to increase student access to exercise facilities and student knowledge of the relationship that exists between exercise and mental health. Indeed, university researchers and educators have skills to develop and trial methods to promote university student mental health; however at present many universities are developing policies without national leadership, guidance or resourcing and support [60]. This highlights a need for partnerships between governments, mental health and higher education service delivers which incorporate data collection on university student mental health.

## Conclusion

This review examined the breadth and outcomes of intervention studies assessing the effects of short-term exercise on mental health in tertiary students. We found that short-term exercise interventions can reduce anxiety like symptoms and anxiety proneness or sensitivity as well as improve mood states in response to an experimentally manipulated laboratory stress test. Positive effects of short-term exercise on additional mental health outcomes in tertiary students were also identified; however there were very few studies available. This indicates that the research regarding the impact of short-term exercise on mental health in tertiary students is currently lacking.

In future research it would be worthwhile exploring the relationship between short and longer-term exercise and how they may relate to each other, in order to maximise the benefits for tertiary students' mental health. The current findings may be useful for consideration in policies and strategies to promote mental health in tertiary students, as although the evidence-base is currently limited, it is preferable that strategies be based on the evidence available, notwithstanding the limitations.

## Data Availability

Data can be obtained from the corresponding author upon request.

## Abbreviations

1RM: 1 Repetition maximum
AET: Aerobic exercise training
AET-high: Aerobic exercise training low intensity
AET-low: Aerobic exercise training low intensity
ASI: Anxiety Sensitivity Index
ASI-R: Anxiety Sensitivity Index-Revised
BDNF: Brain-derived neurotrophic factor
BG: Between group difference
bpm: Beats per minute
CBD: Cannot be determined
E: Exercise
FU: Follow-up
HR: Heart rate
ITT: Intention to treat analysis
LIET: Low intensity exercise training
MOD: Moderate
MHR: Maximum heart rate
mph: Miles per hour
mths: Months
*M*: Mean
m: Minutes
NA: Not applicable
NS: Not stated
*n*: Sample size
PA: Physical activity
PP: Pre-post intervention
PE: Physical education
Reps: Repetitions
RPE: Rating of perceived exertion
RCT: Randomised controlled trial
RET: Resistance exercise training
RET-mod: Resistance exercise training moderate intensity
RET-high: Resistance exercise training high intensity
SBP: Systolic blood pressure
TAU: Treatment as usual
VIG: Vigorous
VO2: Oxygen consumption
wk: Week
WL: Waitlist
yrs: Years
